# To: Efficacy and safety of high-flow nasal cannula oxygen therapy in moderate acute hypercapnic respiratory failure

**DOI:** 10.5935/0103-507X.20200026

**Published:** 2020

**Authors:** Antonio M Esquinas, Habib Md Reazaul Karim

**Affiliations:** 1 Intensive Care Unit, Hospital Morales Meseguer - Murcia, Spain.; 2 Department of Anaesthesiology and Critical Care, All India Institute of Medical Sciences - Raipur, India.

**To the Editor,**

We read with great interest the article “Efficacy and safety of high-flow nasal cannula oxygen therapy in moderate acute hypercapnic respiratory failure” by Yuste et al., where the use of high-flow nasal cannula (HFNC) is evaluated in patients with moderate hypercapnic respiratory failure (HRF).^(1)^ We complement the authors for their exciting work; however, we consider that the conclusions of this study and its results can be commented on from a clinical perspective that in our opinion should be taken into account.

The protocol implemented for HFNC applications needs attention. Although the authors indicate that they used an established protocol, it is essential to know the method of HFNC use in context, specifically, a) if the authors maintained their application during the nighttime^(2,3)^ and b) if the use of the HFNC step with other devices was regulated by some criteria, such as the flow or previous pressures.^(4)^ We think that these considerations can raise the importance of their results and their clinical extrapolation.

Furthermore, although the authors use hypercapnia as a criterion, the patients in their study may have a different etiology of hypercapnia (hypoventilation, increased airway resistance or cardiac insufficiency). This perspective on the use of HFNC can condition the results and the clinical extrapolation of them.^(5,6)^

Lastly, we do not reject the authors’ claim about the efficacy and safety of HFNCs in such patients, but we feel that other aspects of interest, such as time to control of hypercapnia, clinical improvement and successful management of patients with noninvasive mechanical ventilation after HFNC failure, also need to be described and considered. The authors have defined the indication for the use of the HFNC, but selecting one method over another was kept at the physicians’ discretion, which poses an inherent bias. Therefore, even though they had acceptable nonresponders to HFNC therapy, future studies will be required for better acceptance of their results and conclusions.
